# Supplementary material for: Flexible Electrode by Hydrographic Printing for Surface Electromyography Monitoring
Source: Materials (Basel). 2020 May 19;13(10):2339. doi: 10.3390/ma13102339 (PMC7287936; doi:10.3390/ma13102339)
Supplement: Supplementary file 1 [file materials-13-02339-s001.pdf]

Three independent graphs for sEMG recording under each movement are provided as supplement of the revised manuscript.

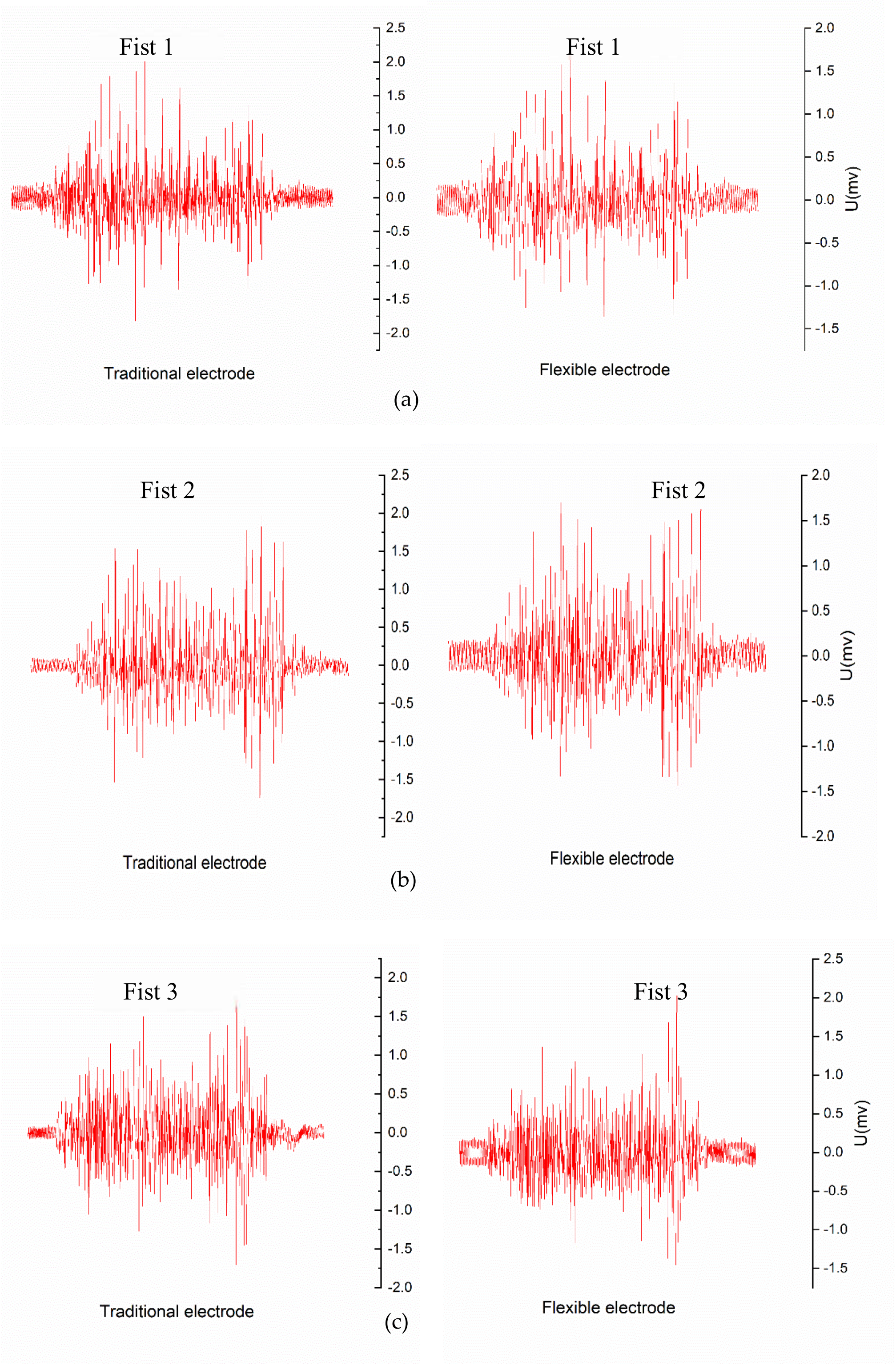

Figure S1. movement of fist (**a**) the sEMG of fist 1 (**b**) the sEMG of fist 2 (**c**) the sEMG of fist 3

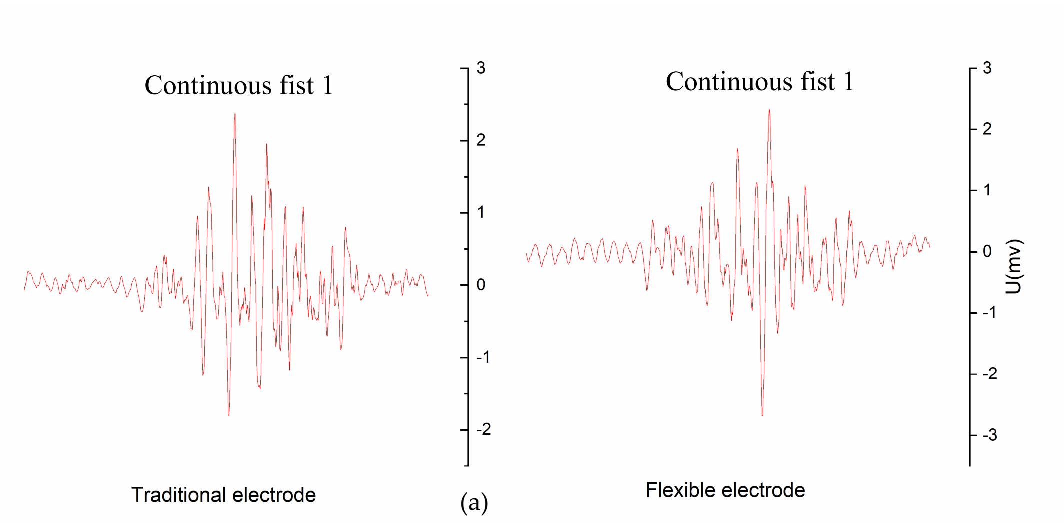

Continuous fist 1
Continuous fist 1
U(mv)
Traditional electrode
Flexible electrode

(a)

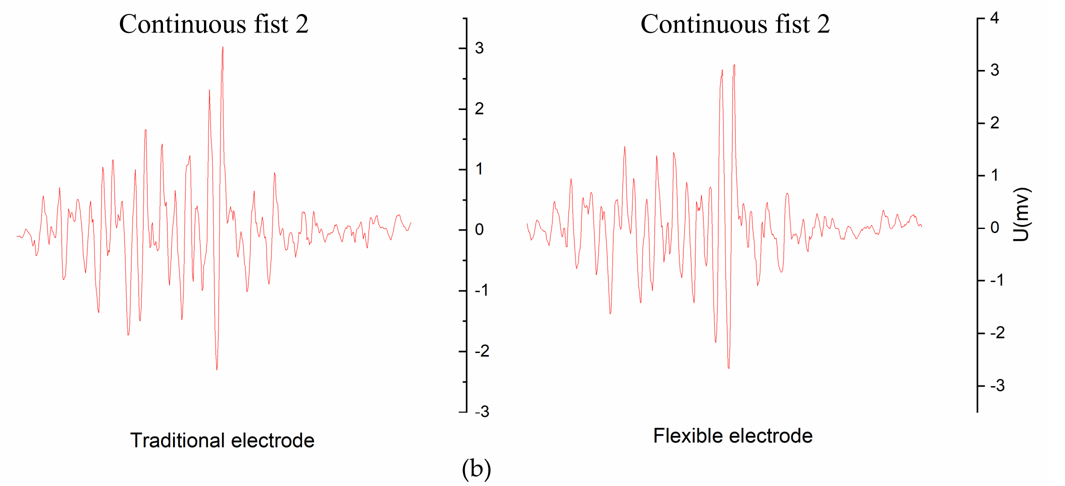

Continuous fist 2
Continuous fist 2
U(mv)
Traditional electrode
Flexible electrode

(b)

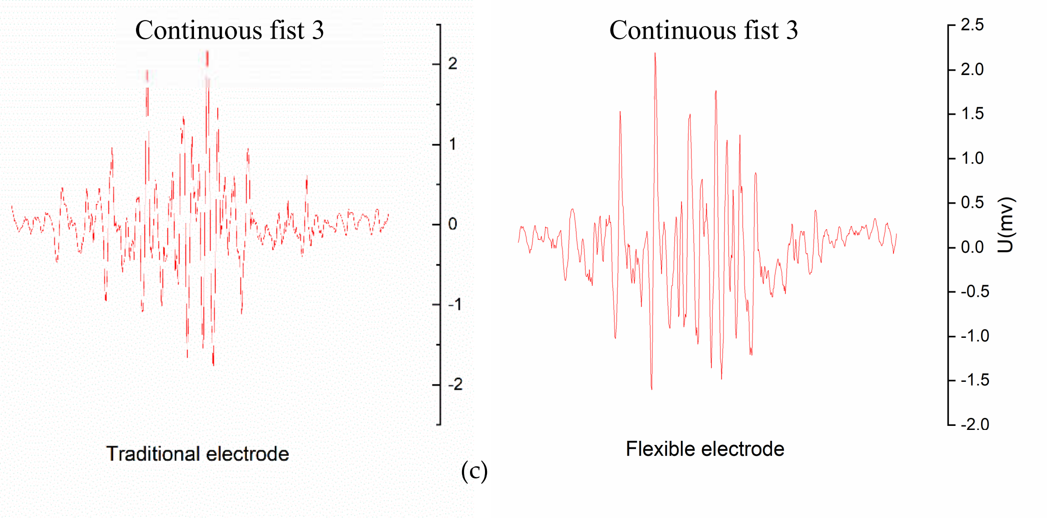

Continuous fist 3
Continuous fist 3
U(mv)
Traditional electrode
Flexible electrode

(c)

Figure S2. movement of continuous fist (**a**) the sEMG of continuous fist 1 (**b**) the sEMG of continuous fist 2 (**c**) the sEMG of continuous fist 3

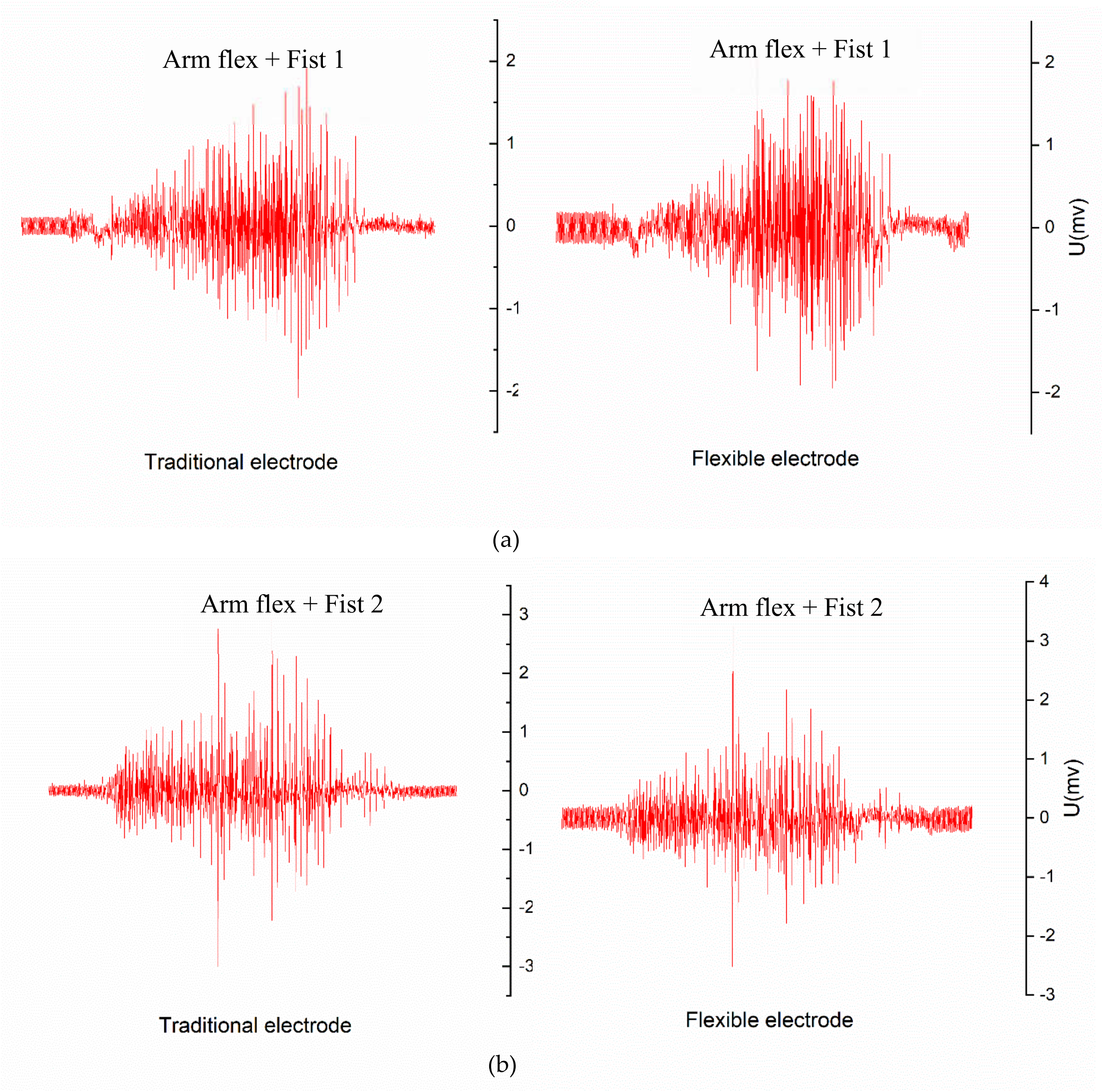

Figure S3. movements of arm flex combining fist (**a**) the sEMG of arm flex combining with fist 1 (**b**) the sEMG of arm flex combining with fist 2.
